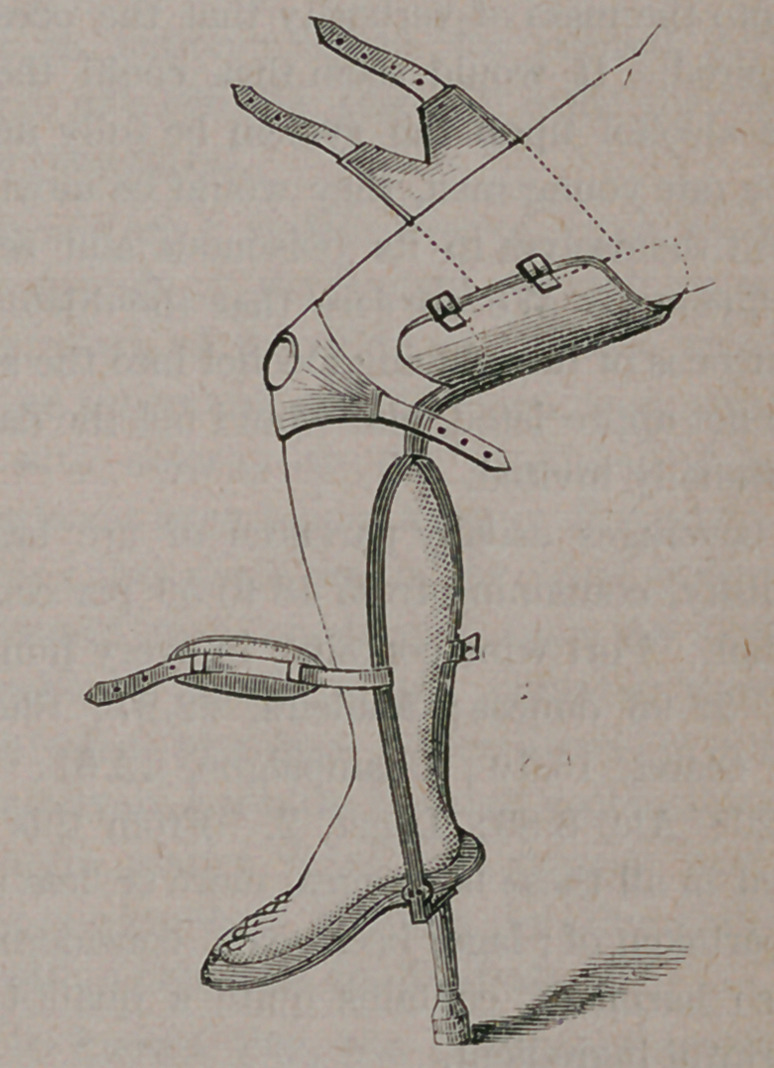# False and Stiff Joints

**Published:** 1877-07

**Authors:** 


The Bistoury.

ELMIRA, N. Y., JULY, 1877.

The BISTOURY is published Quarterly, upon the 1st of April, July, October and January, at Fifty Cents a year in advance.

### FALSE AND STIFF JOINTS.

For the information of several who have written letters of inquiry relative to the construction of apparatus to assist locomotion, in cases of false and stiff joints,—where surgical operations for their cure were undesirable or unattainable by reason of the great distance from a surgeon, we present two cuts illustrative of such apparatus.

The first one exhibits the manner in which a support can be made where a false joint exists in the leg or thigh, from an ununited fracture; for it must be known that it sometimes occurs that fractures do not unite, leaving a false or movable joint at the seat of fracture, so that the limb cannot be made stiff enough to bear the weight of the body. When this occurs, and the patient is unable to secure the services of a competent surgeon and have an operation performed to unite the two ends of the broken bone, an apparatus like the following can be worn, that will give complete control and use of the limb:

It consists of two steel braces, one of which, the outer, terminates in a stout padded band of steel, which is buckled about the hip bones, while the inside one reaches as far as the upper third of the thigh. To these two braces are fastened calf and thigh bands, made of sole leather and moulded to the shape of the limb. They are secured in position by means of buckles, as seen in the illustration. At the ankle, knee and hip joints, are corresponding joints in the two steel braces. This apparatus, when snugly applied, gives good support to the limb and renders its use comfortable.

The reverse of this difficulty is an *anchylosis*, or locked-knee joint, where no motion is possible. By reason of the leg being at an angle with the thigh, with the joint stiff, the limb is too short, requiring some apparatus to give it the required length:

This is accomplished with a well padded trough, of light steel, attached to a strong steel stem, which terminates in two bars below the knee, passing down on each side of the leg, and to the bottom of which is fastened a swiveled steel sole, upon which the foot rests, and to which is attached a staff of the required length, with a rubber bottom, to reach the ground. The weight of the body rests on the side stems, in a vertical line, without giving discomfort to the knee. The knee is kept in position by a knee-cap, and the thigh secured by means of straps, as seen in the engraving.

Those who have not sufficient ingenuity to make their own apparatus, can have them constructed for them, by Geo. Tiemann & Co., 67 Chatham street, New York, to whom inquiries should be addressed for instruction in measurements.

## Figures and Tables

**Figure f1:**
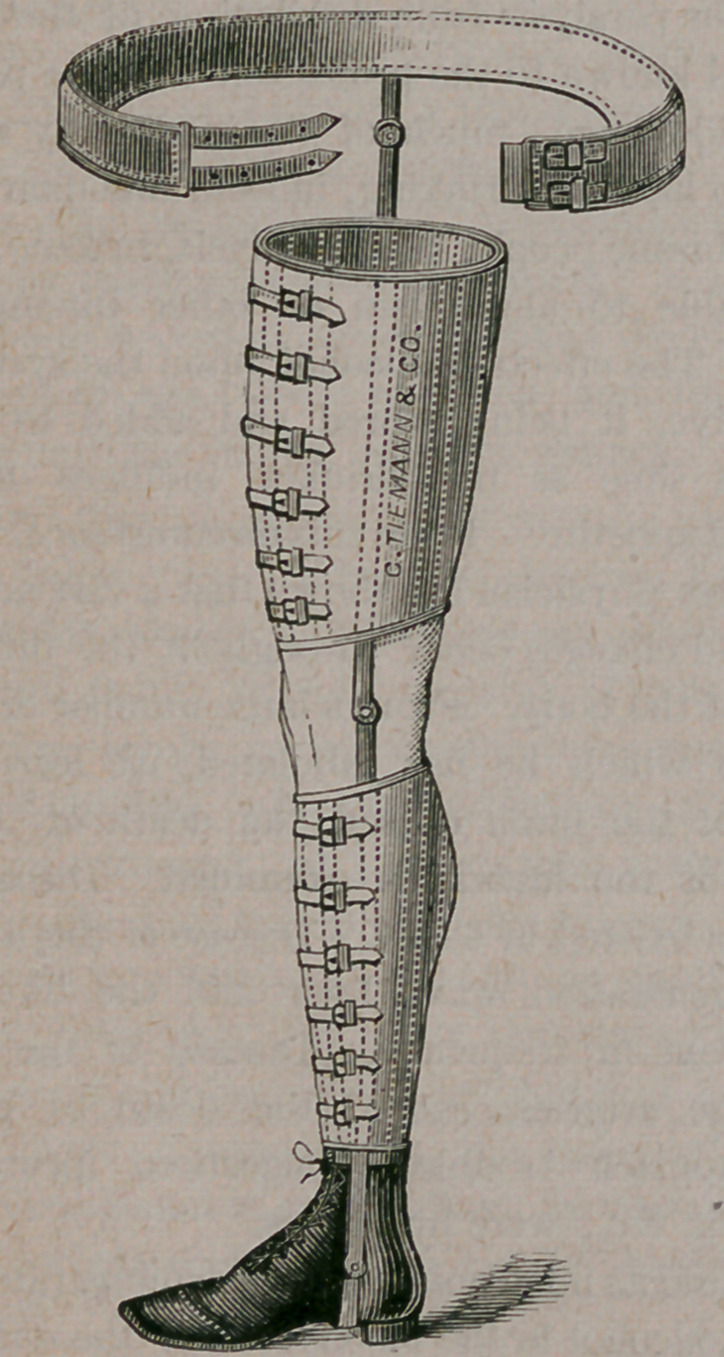


**Figure f2:**